# Lumbar multiunit activity power spectrum during air stepping in the spinal cat: evidence for a flexor-dominated rostrocaudally distributed locomotor center

**DOI:** 10.1152/jn.00310.2022

**Published:** 2023-04-05

**Authors:** Chantal McMahon, David P. Kowalski, Alexander J. Krupka, Michel A. Lemay

**Affiliations:** ^1^School of Biomedical Engineering, Science, and Health Systems, Drexel University, Philadelphia Pennsylvania, United States; ^2^Department of Biology, DeSales University, Center Valley, Pennsylvania, United States; ^3^Department of Bioengineering, Temple University, Philadelphia, Pennsylvania, United States

**Keywords:** central pattern generator, spectral power, spinal cord injury, traveling wave

## Abstract

Clues about the organization of spinal networks responsible for rhythmic motor behaviors have come from the examination of reflex circuitry, lesioning studies, and single-cell recordings. Recently, more attention has been paid to extracellularly recorded multiunit signals thought to represent the general activity of local cellular potentials. Focusing on the gross localization of spinal locomotor networks, we used multiunit signals of the lumbar cord to classify the activation and organization of those networks. We employed power spectral analysis to compare multiunit power across rhythmic conditions and locations and to infer patterns of activation based on coherence and phase measures. We found greater multiunit power in midlumbar segments during stepping, supportive of previous lesioning studies isolating rhythm-generating capabilities to these segments. We also found much greater multiunit power during the flexion phase of stepping than during the extension phase for all lumbar segments. Greater multiunit power at flexion indicates increased neural activity during this phase and is suggestive of previously reported asymmetries between flexor- and extensor-related interneuronal populations of the spinal rhythm-generating network. Finally, the multiunit power showed no phase lag at coherent frequencies throughout the lumbar enlargement indicative of a longitudinal standing wave of neural activation. Our results suggest that the multiunit activity may be representative of the spinal rhythm-generating activity that is distributed in a rostrocaudal gradient. Additionally, our results indicate that this multiunit activity may operate as a flexor-dominant standing wave of activation that is synchronized throughout the rostrocaudal extent of the lumbar enlargement.

**NEW & NOTEWORTHY** We report on the power spectral analysis of multiunit activity (MUA) of lumbar spinal interneurons during a locomotor task. In line with prior studies, we found evidence of greater power at the frequency of locomotion in high lumbar segments and during the flexion phase. Our results also confirm prior observations from our laboratory that the rhythmically active MUA behaves as a longitudinal standing wave of neural activation that is flexor dominant.

## Graphical abstract

**Figure:**
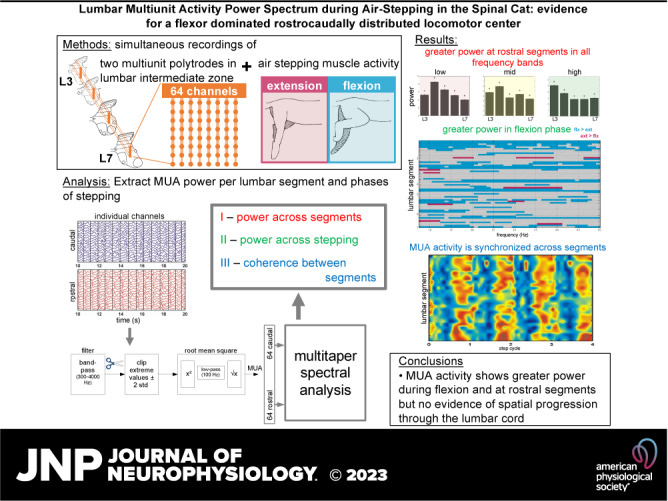

## INTRODUCTION

Multiunit signals are thought to represent the generalized output of neurons localized within an ∼140- to 300-µm radius of the recording electrode (1–3) and are a logical supplement to single-unit firing analysis in neural control studies. In this study, we performed power spectral analysis on the multiunit activity during stepping to explore issues about the neural encoding of locomotion. Power spectral analysis and coherence measures have been used to investigate motor physiology in corticomuscular and corticospinal synchronization (4–8) and spinal motoneuronal/interneuronal activation during fictive locomotion (9, 10). In this study, three power spectral analyses that explored the localization of interneuronal activity during stepping were conducted: *1*) comparisons of rostrocaudal multiunit power to identify the lumbar segments of greatest activity during stepping, *2*) comparisons of flexion and extension phase multiunit power to identify the distribution of neural activity power during rhythmic alternation, and *3*) coherence and phase measures to identify the rostrocaudal activation patterns along the lumbar cord during stepping.

### Localization of Rhythm-generating Centers

Studies of the spinal rhythm-generating centers indicate that the L3–L5 segments are necessary to generate the scratching rhythm in the adult cat (11). By cooling the dorsal surface of the cord and damaging the gray matter of lumbar segments with a heated wire, this study determined that the L3 and L4 segments together, as well as the L5 segment individually, were capable of oscillatory output, while L6 and lower segments were only capable of generating rhythm on their own under certain conditions. Furthermore, lesioning studies in the adult spinal cat show that caudal L3–L4 segments are vital for the rhythmic activation of locomotor output (12). Similar results have been found in many animal models, leading to the recognition of a rostrocaudal gradient of neural rhythmic generation capability along the lumbosacral spinal cord across species (13–18).

### Flexion and Extension Activity during Locomotion

The two-level model of the central pattern generator proposed by McCrea and Rybak (19) was formulated to explain gaps in the half-center hypothesis such as bifunctional muscle activation patterns and nonresetting deletions, whereby motor neuron activity disappears, but flexor and extensor timing is maintained upon the reappearance of the rhythm (20, 21). The first level is described by two excitatory rhythm-generating subunits that set the phasic information for extensor and flexor premotor populations through mutual inhibition between the subunits, the “half-center” model. The second level consists of pattern formation subunits, driven by the rhythm-generating networks of the first level, that shape the activation of the motoneurons. A recent model, the “balanced sequence generator,” argues that the rhythmic properties may actually emerge from the rotational dynamics of a balanced network of sparsely connected excitatory and inhibitory neurons rather than through reciprocal mutual inhibition between flexor and extensor-related interneurons (22).

Inconsistencies in the patterns of drive to flexor and extensor motor populations (23, 24) and other findings (25) led to the idea that the term “half-center” in reference to the rhythm-generating centers was a simplification and that, biologically, each center could operate semiautonomously and not always in a 1:1 reciprocal fashion. Evidence for an asymmetric interaction between flexor and extensor rhythm-generating centers comes from experiments showing that flexor-controlling interneuron populations are inherently rhythmic while extensor populations are tonically active (26, 27). Extensor output is thus thought to be modulated by inhibitory interneuron populations receiving input from phasically active flexor populations (26). More recent evidence for a flexor dominant rhythm-generating half center (28) comes from experimental results in the neonatal mouse fictive locomotion prep (27). The experiments show a strict asymmetry in antagonist motor output where the absence of flexor motor neuron activity was associated with tonic extensor motor neuron activity. Alternatively, an absence of extensor motor neuron activity was associated with no change in the rhythmic behavior of flexor motor activity. The differences in flexor and extensor output during the absence of the antagonist muscle led to the hypothesis that the flexor rhythm-generating population is phasically active and the extensor rhythm-generating population is tonically active.

### Rostrocaudal Activation Patterns along the Lumbar Spinal Cord

Interneuronal activation patterns along the lumbar spinal cord are hypothesized to play a role in the network organization of the neural control of locomotion. Three activation patterns were considered: a traveling wave progression of activation, a longitudinal standing wave of synchronous activation, and a modular organization of nonlinear progression of activation.

The traveling wave is a linear progression of interneuronal activity from rostral to caudal spinal segments that is seen in fictive swimming in the larval zebrafish (29–31) and fictive scratching in the adult cat (32, 33) and suggested during fictive locomotion in the neonatal mouse (9). This traveling wave progression of spinal activation could indicate two organizational schemes of the central pattern generator: either segmentally coupled oscillators of many interconnected central pattern generators or a single forceful distributed central pattern generator (CPG) controlling all oscillators (31). The latter theory could attribute the traveling wave to the physical separation of spinal segments as the increased distance from the CPG epicenter causes conduction delays to more caudal segments (34).

The longitudinal standing wave is characterized by synchronous activation of locomotor-related interneurons as a coherent network distributed throughout the lumbar enlargement. AuYong et al. (35, 36) provided evidence supporting a longitudinally distributed network within the intermediate zone of the spinal air-stepping cat in which single-unit and multiunit activity of interneurons from L3 to L7 were concurrently activated during stepping and temporally correlated to the ipsilateral swing to stance transition period during air stepping. More recently, spontaneous activity of cord dorsum potentials in the anesthetized cat from lamina III–IV has demonstrated synchronous activity, bilaterally distributed throughout the extent of the lumbosacral cord in both the intact and the acutely spinalized cat (37). While these longitudinal standing activations patterns of cord dorsum potentials were spontaneously active and not associated with a rhythmic behavior, they are hypothesized to represent tightly coupled networks of spinal interneurons involved with the modulation of information transmission from sensory systems to motor systems.

Finally, the modular organization model hypothesizes that the central pattern generator is made up of nonlinearly coupled oscillators. Recent evidence of such patterning is seen in human and cat motor pools (38, 39) where motor neuron activation during stepping oscillates between rostral and caudal spinal segments. A similar theory comes from the dynamics of multiple synfire chains (40) in which premotor modules are connected across multiple segments that do not fire in a linear spatiotemporal fashion due to chains of inhibitory neurons that increase in activity through synchronization.

The multiunit power analyses presented in this article address those three issues regarding the neural control of locomotion by specifically investigating *1*) rostrocaudal differences in the interneuronal activity during locomotor behavior, 2) differences in neural activation levels during opposing phases of stepping, and *3*) patterns of interneuronal population activity along the lumbar cord.

## MATERIALS AND METHODS

### Animals and Experimental Procedures

Five adult domestic short hair female cats (2.4–2.9 kg) were used in this study. All animal care and procedures were approved by the Institutional Animal Care and Use Committee of Drexel University and were performed according to National Institutes of Health guidelines. All animals followed the same experimental protocol. First, a T11/T12 spinal transection was performed 22–23 days before the recording session to potentiate the air stepping induced by clonidine administration during the terminal procedure (41). Spinal transection and postprocedure care followed the laboratory standard procedures (41, 42). No locomotor training was provided to the animals at any point. On the day of the terminal recording experiment, three sets of surgical procedures were performed on the anesthetized animal: *1*) a laminectomy that exposed the lumbar cord, *2*) bifilar electromyogram (EMG) electrodes were implanted into seven muscles of each hindlimb, and *3*) a midcollicular decerebration preceded the discontinuation of anesthesia.

### Surgical Procedures Before Recording Session

Consistent with other multiunit recording experiments in our laboratory (35, 36, 41), animals were initially injected with atropine (0.05 mg/kg, IM) and anesthetized with isoflurane (1.5–3.5% in oxygen) supplied through an endotracheal tube. Heart rate, blood pressure, end-tidal CO_2_, tidal volume, arterial oxyhemoglobin saturation, respiration rate, and temperature were monitored and recorded every 15 min. Intravenous fluids were administered (20 mL/h) throughout the terminal procedure and dexamethasone (2 mg/kg, IV) was given before the laminectomy to reduce spinal swelling. A spinal laminectomy removed the exposed spinous processes from sacral segment one rostrally toward lumbar segment three and the surrounding bone, leaving the transverse processes of each segment intact and exposing the spinal cord from segments L2 to L7.

### EMG Electrodes Implant

Muscles of the upper and lower hindlimbs were exposed with two incisions. Seven muscles of each hindlimb (sartorius anterior, biceps femoris anterior, biceps femoris posterior, vastus lateralis, gastrocnemius medialis, soleus, and tibialis anterior) were implanted with bifilar electrodes constructed with insulated multistrand stainless steel wires (AS 633; Cooner Wire, Chatsworth, CA). The electrodes were implanted into the body of the muscle and secured onto the fascia with sutures. Proper electrode placement was verified by stimulation of the electrode and observation of the resulting muscle twitches. Incisions were closed with sutures. A stimulating cuff electrode was implanted around the sciatic nerve to identify motor neurons that backfired at a short latency in response to electrical stimulation of the nerve.

Following the laminectomy and EMG electrode placement, the animals were transferred to a stereotaxic frame where the spinal vertebrae were securely clamped to the frame. Trunk skin flaps were used to form a mineral oil pool that prevented desiccation of the cord following the opening of the dura. Roots were identified and used as anatomical landmarks of the lumbar segments. The pia was opened at planned recording sites to ease electrode insertion and prevent dimpling of the cord. A midcollicular decerebration was performed, and anesthesia was discontinued, as isoflurane has been shown to disrupt the activity of the spinal locomotor circuitry (43).

### Extracellular Recording Procedures

At 1 h postdecerebration, clonidine (500 µg/kg) was administered intravenously to prime air stepping (44). Once air stepping was controllably inducible through manual pinching/rubbing of the base of the tail and perineal area, experimental recording sessions began. All of the recording trials were conducted on the right side of the spinal cord. Two 64-site microelectrode arrays (model A8x8-5mm-200-200-177, Neuronexus, Ann Arbor, MI) were inserted at or near the dorsal root entry zone to an approximate depth of 3,000 µm in two lumbar segments. The planar eight shaft arrays were inserted sagitally, i.e., in the rostrocaudal direction, so that the recording sites covered a range of 1,450 µm rostrocaudally and 1,450 µm dorsoventrally (from ∼1,500 to 3,000 µm deep) for each array. In our first experiment, one electrode array was statically placed in L7 while the other array was successively placed in rostral segments L3 to L6. However, low neuronal yield at the L7 site required adaptation of the protocol for the remaining four experiments to static placement of one array in L3, while the other array was successively placed caudally in segments L4 to L7.

The recording trials obtained for each pair of spinal locations consisted of the following: *1*) air-stepping trials involving a 5-s resting state followed by 50 s of air stepping (containing 40–60 step cycles) and a 5-s rest period, *2*) control rest trials (no perineal stimulation), and *3*) sciatic nerve stimulation trials (current amplitude at 1.3 times motor threshold, 1–2 Hz, 60 s, biphasic pulses with a 100-µs duration for each phase) at rest. The rest and sciatic stimulation control trials as well as four to six air-stepping trials were executed at each set of unique recording electrode location pairs.

Extracellular neural activity and muscle EMGs were recorded with a Tucker-Davis Technologies RZ2 system including 128 channels for recording multiunit activity and 15 analog channels for 14 EMGs and 1 sciatic stimulus recording. Extracellular voltages were conditioned through a PZ2-128 preamplifier (Tucker-Davis Technologies, Alachua, FL) and bandpass filtered (300–4,000 Hz for single and multiunit data) before being sampled at 24 kHz for offline analysis. EMG voltages were amplified and bandpass filtered (10–5,000 Hz 4th-order Butterworth, 10 K gain, Differential Amplifier Model 1700, A-M systems Inc., Carlsborg, Wa) before being sampled at 12 kHz. Reference and ground were tied to a bone screw in the skull.

### EMG Data Processing

All data processing and analysis for neuronal and EMG recordings were performed using software written in MATLAB (The Mathworks, Natick, MA).

Raw EMG voltages were high-pass (4th-order elliptical, 100-Hz cutoff frequency, zero phase) filtered, full-wave rectified, and low-pass (4th-order elliptical filter, 15-Hz cut-off frequency, zero phase) filtered to develop linear activity envelopes. EMG burst onsets and offsets were identified with an algorithm based on the generalized likelihood-ratio test (45). The onsets were used to determine the start and end of the step cycle as well as the flexor and extensor phases of gait. A step cycle was defined as right soleus (ankle extensor) onset to consecutive right soleus onset.

Five criteria (35, 36) were used to test the quality of the stepping in terms of both left-right limb alternation and flexor-extensor alternation. Details of the methods and results about the quality of stepping can be found in McMahon et al. (41).

### Neural Signals Data Analysis Overview

**Figure 1.**
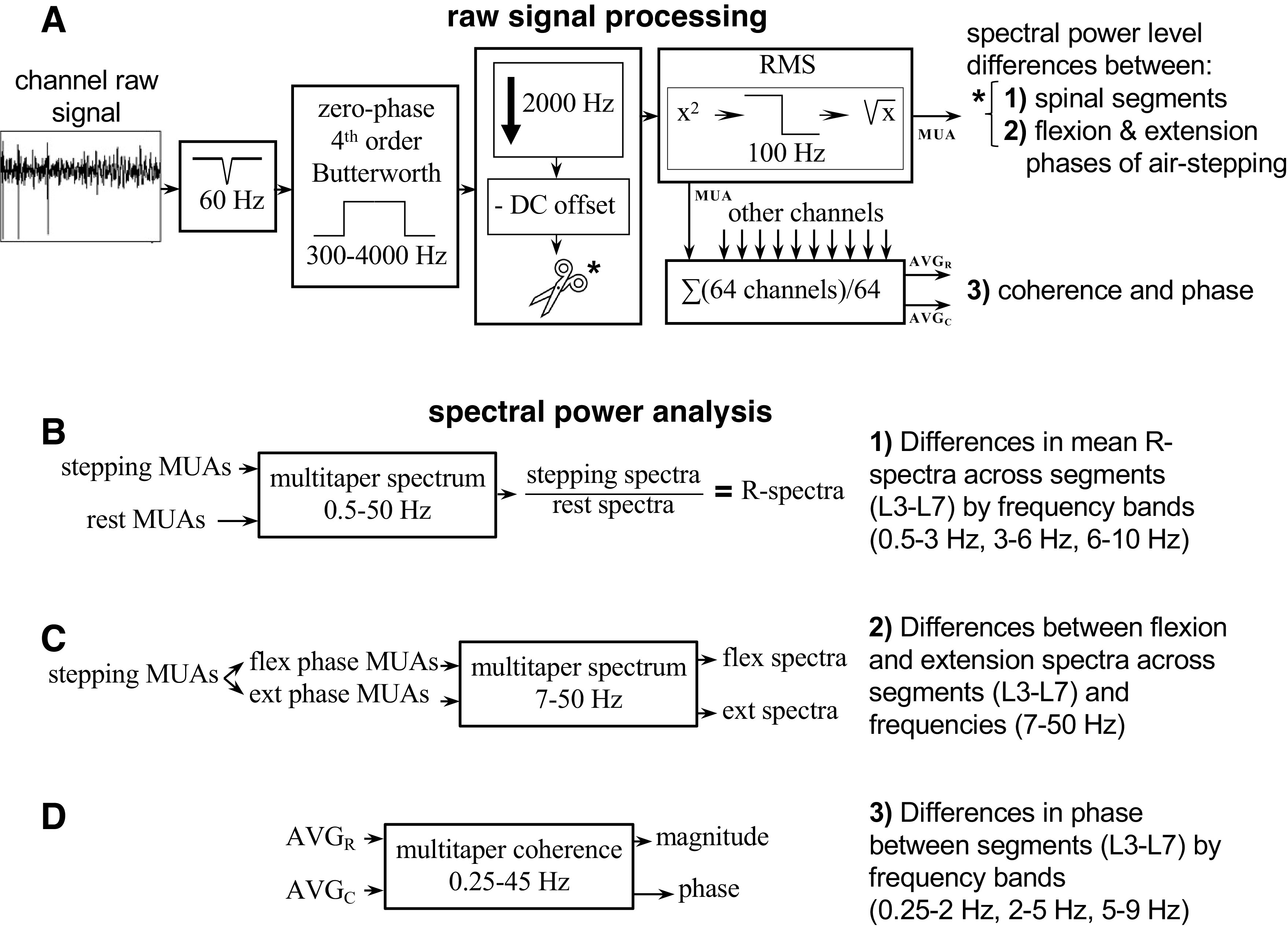
*A*: raw signal processing for each multiunit electrode recording site. The signal is comb (60 Hz) and band-pass (300–4,000 Hz) filtered before being downsampled to 2,000 Hz. DC offset is then removed, and large units are clipped (for the spectral power level differences, *analyses 1* and *2*) before the signal is root mean squared (RMS; low-pass cut-off of 100 Hz). *B* and *C*: the processed signal from each channel is then used to analyze the difference in spectral power during stepping normalized to the resting spectra between spinal segments (*B*) or between spectral power during the flexion and extension phases of air stepping (*C*) using multipaper spectra of the multiunit activity (MUA) signal. *D*: the average signals from the 64 sites at the rostral and caudal electrodes (AVG_R_ and AVG_C_ in *A*) are used to analyze the differences in phase between segments using multitaper coherence analysis.

Three analyses were used to compare various features of the multiunit activity in the lumbar spinal cord, *1*) multiunit relative spectral power analysis was used to compare power level between lumbar segments, *2*) multiunit spectral power analysis was used to compare power during flexion and power during extension, and *3*) multiunit coherence and phase between multiunit activity (MUA) signals (64 channels averaged per lumbar segment) were used to relate the timing of MUA activity between lumbar segments (see Fig. 1).

### Multiunit Envelope Processing

Multiunit spectral analysis was performed for trials with the greatest number of consecutive steps per recording location. Standard multiunit activity processing methods were used (35, 46). For each channel within an electrode array, a comb filter was applied to remove 60 Hz (and harmonics) of noise. The signal was then zero-phase bandpass filtered with a 4-pole Butterworth filter from 300 to 4,000 Hz. The multiunit signal was downsampled to 2,000 Hz, and the average potential amplitude per signal was subtracted from each channel to remove DC offset (47). To remove large noise artifacts present during some stepping trials, signals that surpassed the 99.99th percentile of the signal amplitude were removed for a 6-ms window surrounding the artifact peak and replaced with the mean signal amplitude (see Fig. 1*A*).

When analyzing spectral power differences between *1*) lumbar segment and *2*) extension and flexion phases, we removed the bias of large amplitude action potentials per channel by clipping each signal at 50 μV and replacing extreme values with a linear interpolation of the surrounding signal (35) before the root mean square procedure. For all three analyses listed above, each channel’s multiunit signal underwent a root mean squared process (square signal, low-pass filter at 100 Hz, square root signal) (46). The coherence and phase analysis *3*) then involved averaging the multiunit envelope across the 64 channels of a single electrode array (5), resulting in two composite multiunit waveforms per trial, one for each lumbar segment recording (AVG_R_ and AVG_C_ of Fig. 1, *A* and *D*).

To test that the increased power in lower frequencies was of biological relevance and not an artifact of the root mean squared procedure, we compared the power, coherence, and phase results between electrode arrays for both rest and stepping conditions. The multiunit envelope process was performed for 20 s of rest at the same locations and we removed the DC offset, averaged the signals across the 64 sites per array, and performed the same root mean squared preprocessing as the stepping multiunit signals. Both the highly coherent frequencies during rest and stepping as well as the duration of consecutive significantly coherent frequencies were considered.

### Comparisons of the Multiunit Power Spectral Density per Lumbar Segment

The two air-stepping trials with the greatest number of steps at each spinal recording location were chosen for this analysis. Multiunit activity at rest was taken from the same channel as the MUA air-stepping trials. The first 18 s of both stepping and rest trials were used in this analysis. Rest trial multiunit signals were treated as the MUA during stepping trials. Analysis was performed per subject, initially, and then grouped across subjects according to the lumbar segment from which they were recorded.

Multitaper spectral analysis was performed with a time-bandwidth product of 4 and 7 tapers, a window size of 18 s, and covered the frequency band from 0.5 to 50 Hz (Chronux package; Refs. 48–51). To compare power across lumbar segments and subjects, the air-stepping multiunit power spectrum was divided by the corresponding rest multiunit power spectrum per channel to give a normalized, unitless power spectrum [R-spectrum of Burns et al. (1)] as shown in the following equation:

$$\mathrm{R(0}.5\text{–}50\text{ Hz}\mathrm{)=}\frac{\mathrm{stepping MUA power spectrum}\;\mathrm{(0}.5\text{–}50\text{ Hz})}{\mathrm{rest MUA power spectrum (0}.5\text{–}50\text{ Hz})}$$

Statistical analysis was used to test the difference in mean relative power per lumbar segment using a one-way ANOVA across five lumbar segments (L3–L7). Comparisons between relative segmental power were broken down by frequency bands (0.5–3 Hz, 3–6 Hz, and 6–10 Hz) that were selected due to peaks in spectral power for the low and high bands that were consistent across subjects (Fig. 1*B*). While there were significant differences in multiunit power per lumbar segment above 10 Hz, the power during rest was equitable or greater than the power during stepping in these frequencies for some lumbar segments. Therefore, the frequency band above 10 Hz was not considered in our results. A Tukey multiple comparisons test (Benjamini-Hochberg multiple corrections, α = 0.005; Ref. 52) was used to detect differences in mean multiunit power between the five lumbar segments.

### Comparisons of Flexion and Extension Phase Multiunit Power Spectral Density

Windows of multiunit activity for one flexor muscle’s bursts and one extensor muscle’s bursts (from the bursts’ onset to the average burst duration) were taken for steps fulfilling the criteria listed below. This analysis resulted in two datasets per electrode array: the first data set applied to the MUA during the extensor burst for all channels and steps and the second applied to the MUA during the flexor burst for all channels and steps (Fig. 1*C*).

Multitaper power spectral estimation was performed for a frequency range from 7 to 50 Hz for a time-bandwidth product of two and three tapers (Chronux; Ref. 48). The multiunit power per stepping phase could not be resolved for frequencies less than 7 Hz due to the short temporal duration of the flexor and extensor phases of stepping. We used a two-group hypothesis test (49) to detect significant differences between the power spectra of multiunit activity during flexion and extension. This test is unique in that it is applicable to unequal sample sizes (in our case the extensor burst duration differed from the flexor burst duration within a trial) and the jackknife estimates of variance of the test statistic do not assume a normal distribution of the data (49). Compensation for bias in this analysis is twofold; first, Jackknife variance estimates allow for the detection of large amounts of non-Gaussian variance among power data across frequencies. Therefore, large variances in data were excluded from consideration for significance when the Jackknife estimated variance of the spectra was greater than 5 (49). Second, due to the decreased frequency resolution introduced by multitaper estimation for a time-bandwidth product of 2, we only considered instances where one condition was significantly greater than another for more than a 4 Hz bandwidth. The Benjamini-Hochberg multiple comparisons false detection rate calculation α = 0.05/(2 × frequencies × channels) was used to compensate for multiple comparisons across 43 frequencies and 64 channels (52–54).

To be considered for analysis, an individual step’s duration had to fall within ±10% of the mean step duration for the trial and the flexor onset phase had to be within ±10% of the mean flexor onset phase for the trial. Step duration was defined as the time difference between the right extensor muscle onset to consecutive right extensor onset. The flexor onset phase was defined as the (right flexor onset time − right extensor onset time)/step duration. Only trials that contained at least 10 steps meeting these criteria were included for analysis.

### Multiunit Coherence and Phase Analysis Used for Detecting the Progression of Rostrocaudal Activation

Coherence and phase between multiunit signals of each lumbar segment were calculated for at least 10 s of stepping (6, 55). Multitaper spectral analysis was performed for each coherence and phase measures with a time-bandwidth product of five and nine tapers for a bandpass frequency from 0.25 to 45 Hz and included jackknife error estimates (α = 0.05, mtcoherencyc; Ref. 48) (see Fig. 1*D*). To be considered highly coherent, MUA coherence had to be greater than the 95% confidence interval and the coherent frequency band’s width had to be greater than 0.37 Hz (24 consecutive samples) (5, 56). Coherence and phase measures were analyzed by frequency band: 0.25–2 Hz (low), 2–5 Hz (medium), and 5–9 Hz (high). While there was some significant multiunit coherence between segments above 9 Hz, the resulting significantly coherent frequency bands were infrequent and inconsistent and therefore not considered for analysis.

The multiunit coherent phases between segments (L3–L4, L3–L5, L3–L6, and L3–L7) were grouped per frequency band (low, middle, and high) across all subjects. A one-way ANOVA was used to test for a difference in mean phase between segments within the same frequency band. Finally, Tukey’s multicomparison test was used to calculate the significant mean phase differences between segments for each frequency band (α = 0.05).

## RESULTS

### Summary

In total, 36 air-stepping trials and 18 rest trials were analyzed across 4 subjects, with an average of 9 ± 1 air-stepping trials per subject. Power analysis required 10 s of continuous stepping, which one animal did not perform on a consistent basis; this animal was therefore excluded. Twenty-five unique recording locations were sampled across all subjects used with 18 unique pairs of rostrocaudal locations. While the recording locations sampled varied per subject, each lumbar segment was recorded at least eight times (L3 = 24, L4 = 14, L5 = 8, L6 = 16, and L7 = 10) and when considered per unique location, recorded at least four times (L3 = 4, L4 = 5, L5 = 4, L6 = 8, and L7 = 4). The dorsoventral recording depth spanned from 1,560 ± 217 μm to 2,960 ± 217 μm. Single-unit activity results for those animals are reported in McMahon et al. (41).

**Figure 2.**
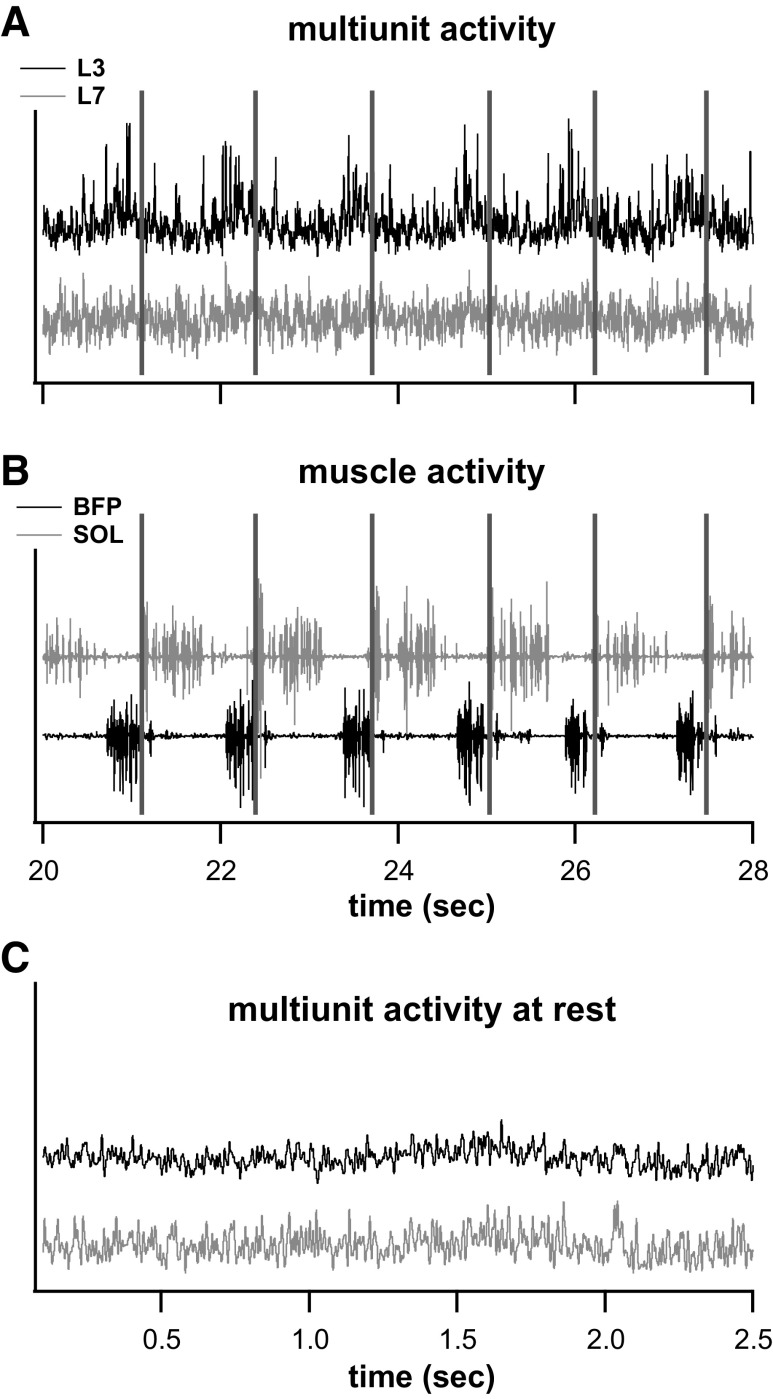
*A* and *B*: multiunit activity (MUA) envelope and electromyogram (EMG) activity over 6 air-stepping steps. *A*: examples of MUA averaged per electrode array for the L3 (top/black) and L7 (bottom/gray) segments. Modulation in MUA was more pronounced for the L3 spinal segment. *B*: corresponding extensor (top/gray) and flexor (bottom/black) EMG activity. Vertical bars indicate extension onset. BFP, biceps femoris posterior; SOL, soleus. *C*: MUA averaged as in *A* but before the onset or air stepping, i.e., at rest. There was no modulation in MUA activity and no EMG activity (data not shown) at rest.

**Figure 3.**
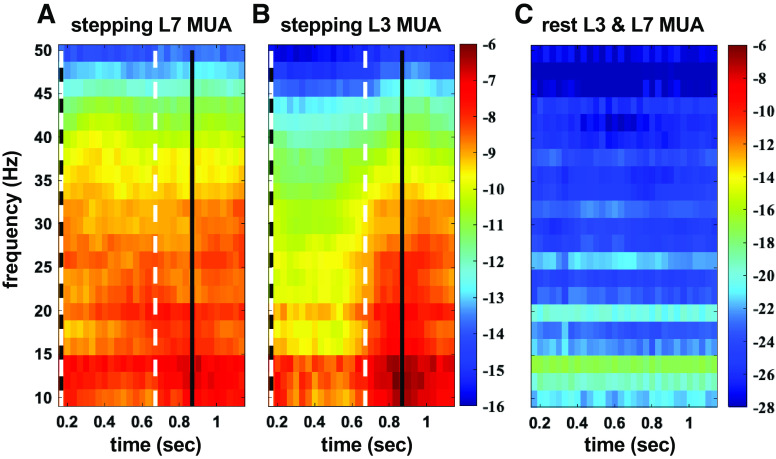
Time-frequency power spectra of the multiunit activity of Fig. 2. *A*: spectral averaged multiunit activity (MUA) of the electrode array in L7. *B*: spectral averaged MUA of the electrode array in L3. These spectra were averaged across the steps of an air-stepping trial. The white dashed vertical lines represent the average extensor onset (∼0.15 s) and extensor offset (∼0.65 s). The solid black vertical lines represent average flexor onset (∼0.82 s) and flexor offset (∼0.15 s of the consecutive step). *C*: spectral averaged multiunit activity over 128 channels of arrays during 20 s of rest at the same L3 and L7 locations. Panel shows that the root mean square process introduces some higher power at low frequencies; however, the power level introduced is negligible compared with the power during stepping (*z*-axis unit, dB).

Figures 2 and 3 show the multiunit activity during a single air-stepping trial. Figure 2 shows the multiunit activity averaged over the electrode array per lumbar segment as well as the corresponding EMG activity across six steps. Figure 3 shows the average multiunit activity spectral power (in dB) per array across a step for the trial in Fig. 2. The modulation in the L3 multiunit activity during the step cycle in Fig. 2*A* is matched by the increased power during flexion in Fig. 3*B*.

Finally, our control for the low-frequency power introduced by the root mean square processing of multiunit activity is shown in Fig. 3 *C*. While there is greater power in the low frequencies (compared to higher frequencies) during rest, the power at all frequencies is negligible. Additionally, when multiunit rest signals underwent coherence measures between lumbar segments locations, the coherence during rest rarely went above the 95% confidence interval for any frequency and the lower 95% confidence interval of the jackknife estimate never went above the 95% confidence interval for significant coherence.

### Comparisons of the Multiunit Relative Spectral Power between Lumbar Segments

The number of trials at each lumbar segment and the spinal lumbar segments used for the analysis of the MUA spectral power are shown in Table 1. Overall, there were at least eight MUA air-stepping trials analyzed per lumbar segment.

**Table 1.** Number of trials at each lumbar segments used for analysis of the MUA spectral power

	Information			
Subject	No. of Trials	No. of Steps	Average Flexion Burst, s	Average Extension Burst, s
*1*	10	34 ± 13	0.39 ± 0.14	0.48 ± 0.13
*2*	6	22 ± 8	0.18 ± 0.05	0.33 ± 0.06
*3*	7	36 ± 13	0.32 ± 0.05	0.44 ± 0.1
*4*	6	19 ± 7	0.29 ± 0.06	0.55 ± 0.05

MUA, multiunit activity.

**Figure 4.**
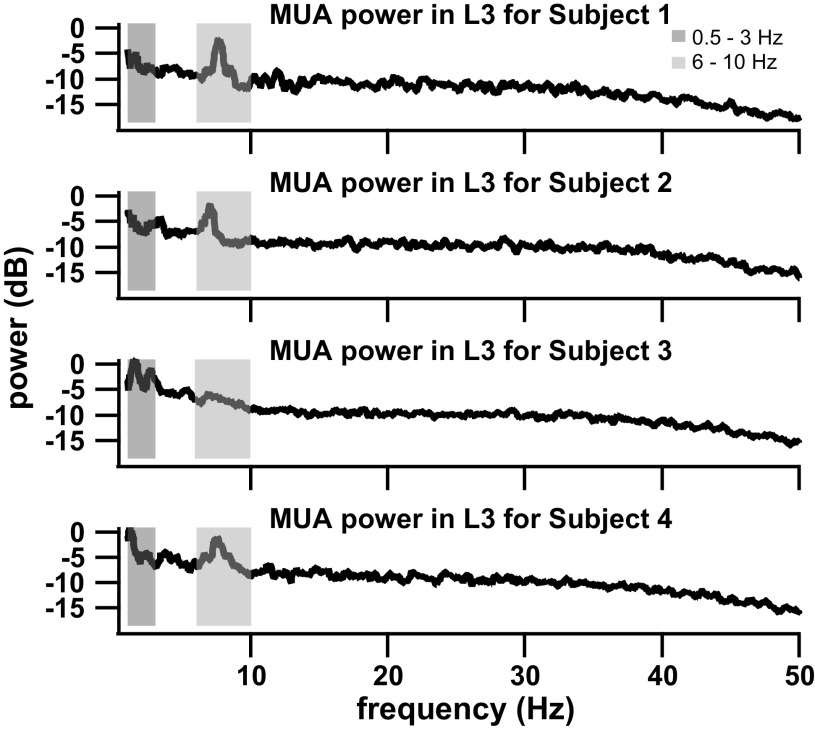
Multiunit power spectra during stepping in the L3 segment for each subject. Power spectra show distinct peaks in the 0.5- to 3-Hz and 6- to 10-Hz frequency bands, and those peaks were repeatable across subjects. These peaks in multiunit activity (MUA) spectra were not seen during rest.

**Figure 5.**
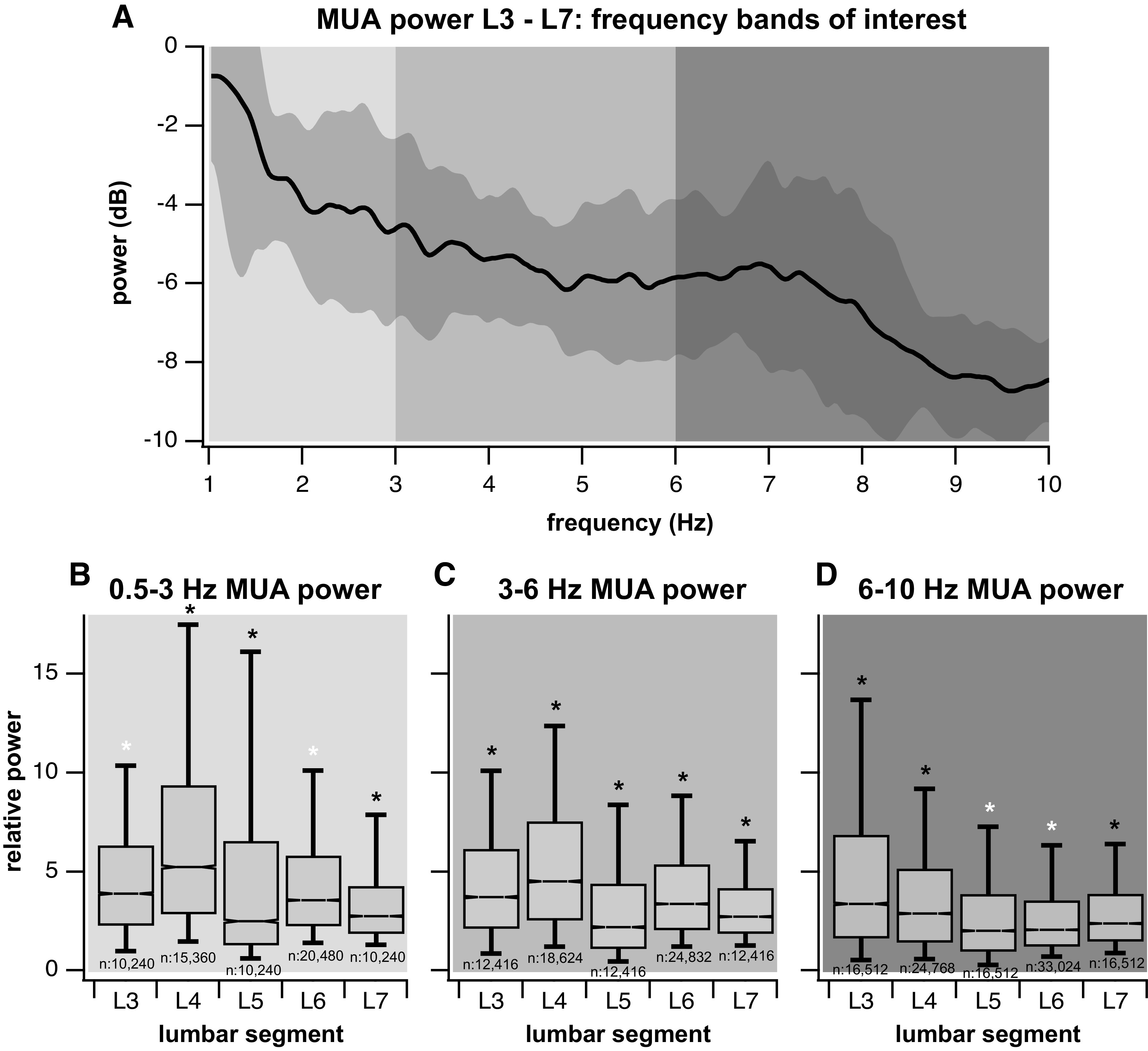
Comparisons in multiunit activity (MUA) power between lumbar segments for 3 frequency bands. *A*: the averaged power spectrum for all subjects and lumbar segments (with 95% confidence interval of the averaged power over the four subjects indicated by dark shading around the mean). Peaks in MUA power are seen around 1 Hz and between 6 and 9 Hz. The background shadings correspond to the frequency bands shown in at the *bottom*. *B*: the lowest frequency band from 0.5 to 3 Hz had the greatest MUA power in L4. The MUA power in L5 was also greater than in segments, L3, L6, and L7. Black asterisks indicate statistical significance differences in mean multiunit power from other lumbar segments (ANOVA post hoc Tukey multiple comparisons test with Benjamini-Hochberg multiple corrections, α = 0.005). Segments with white asterisk did not have significantly different MUA power from each other. *C*: 3- to 6-Hz MUA power was also the largest in L4. However, the power in L5 decreased compared to the low frequency MUA power per lumbar segment. *D*: the MUA power in the high-frequency band was greatest in L3, decreasing slightly in L4 and decreasing further in L5, and staying low in L6 and L7. Not pictured is the distribution of lumbar segment multiunit power from 10 to 50 Hz, it displayed a similar rostrocaudal gradient to the 6- to 10-Hz distribution where power in L3 was greatest and decreased caudally. The power at this frequency band was no different during stepping than rest and therefore is not pictured. Whiskers in *B*–*D* indicate the 9th and 91st percentiles. The number of points is indicated below each of the box plot.

Small differences in power content per lumbar segment were detected across subjects (see Fig. 4) and therefore, results were combined for all subjects (Fig. 5). In the low**-**frequency range (0.5–3 Hz), the MUA power showed a peak around 0.75–1.25 Hz in all lumbar segments (Figs. 4 and 5). Comparisons across segments showed that the MUA power in L4 was much greater than in all other segments in the low**-**frequency range (Fig. 5*B*). Additionally, the MUA power in L5 was also significantly greater than in the L3, L6, and L7 segments.

In the midfrequency range (3–6 Hz), the multiunit power spectrum did not have any peaks in any lumbar segment (Figs. 4 and 5). Again, L4 showed much greater MUA power than the remaining segments (Fig. 5*B*). L3 also showed greater power than L5, L6, and L7. The power in L5 was the lowest MUA power across segments and comparable to the power in L7.

The high-frequency band from 6 to 10 Hz displayed a significant peak within this range for many of the subjects and lumbar segments as seen in the broad peak of the averaged power spectra in that band for all subjects (Fig. 5*A*) and in the distinct peaks within individual subjects (L3 example, Fig. 4). The location of the peak varied slightly between 6 and 8 Hz across subjects (Fig. 4). The comparisons in MUA power between segments showed the greatest power in the L3 segment, coupled with a decrease for the more caudal segments, and with power in L4 being significantly greater than in L5, L6, and L7. The power at the higher frequencies (10–50 Hz) was comparable between stepping and rest MUA and did not display any peaks within this range. Over the 0.5- to 10-Hz frequency band, the relative power differences between segments were consistent with power decreasing for the more caudal segments.

### Comparisons between Multiunit Spectral Power during the Flexion and Extension Phases of Stepping

Twenty nine air-stepping trials were used in this analysis. There were on average 29 ± 13 steps per trial. The average flexor burst duration was 0.31 ± 0.12 s and the average extensor burst duration was 0.45 ± 0.12 s (see Table 2 for more details).

**Table 2.** Summary of the data used for comparing multiunit spectral power during the flexion and extension phases of stepping

	Segment				
Subject	L3	L4	L5	L6	L7
*1*	2	8	0	2	6
*2*	2	0	2	4	2
*3*	2	2	2	6	0
*4*	2	2	4	4	2
Total	8	12	8	12	10

**Figure 6.**
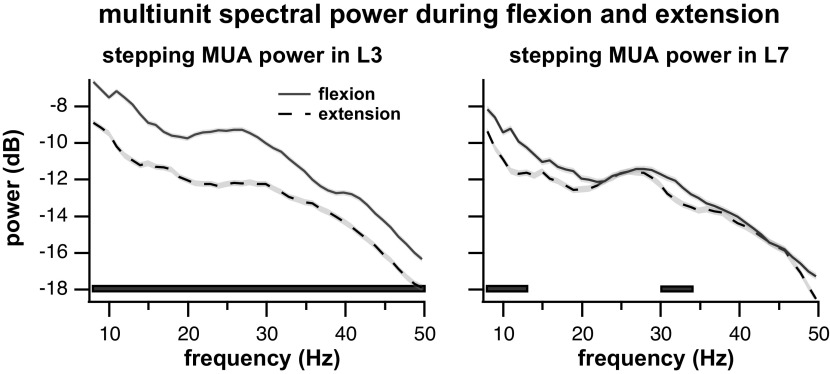
Two examples of power spectra of multiunit signals during flexion and extension in different lumbar segments. These are the same data as seen in Fig. 3, *A* and *B*. Estimates are trial averaged over muscle bursts and 64 channels per array. Estimates include 95% confidence interval of Jackknife variance (shaded area around the lines). Bars along the *bottom* indicate sections of statistically significant differences in power spectra as determined by the two-group test and Benjamini-Hochberg test of multiple comparisons; bar line type indicates which spectrum was higher. Spectra are smoothed with a 3-point moving average filter for visualization. MUA, multiunit activity.

Two examples of multiunit power spectra during flexion and extension are presented in Fig. 6 These data were recorded in the same air-stepping trial over two multiunit electrode arrays placed in L3 and L7 (Fig. 6, *A* and *B*, respectively). The power difference between MUA during flexion and extension in the L7 segment was negligible over most frequencies (Fig. 6*B*) while the multiunit power during flexion was much greater than during extension across all frequencies for the L3 segment (Fig. 6*A*).

**Figure 7.**
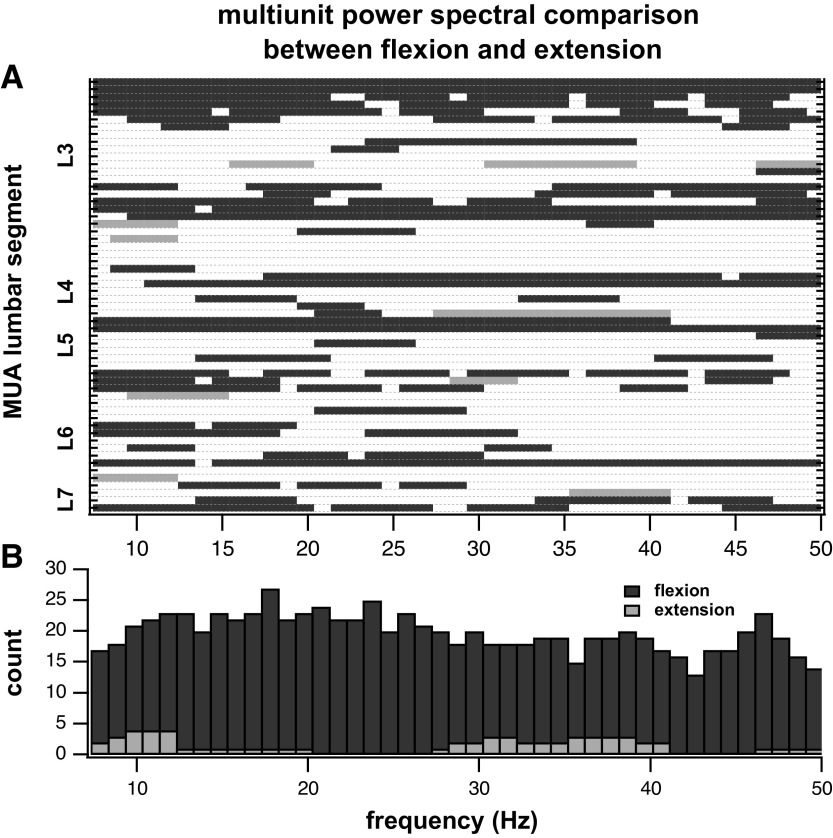
Comparisons between the multiunit power spectral density during flexion and extension phases of air-stepping trials. *A*: rows are ordered according to the lumbar segment from which they were recorded. Gray bars represent frequencies where the multiunit power during extension was significantly greater than during flexion. Black bars represent frequencies where the multiunit power during flexion was greater than during extension. White areas represent frequencies where there was no significant difference in multiunit activity (MUA) power between flexion and extension. *B*: summed count of the top panel rows representing counts of the stepping phase with the larger MUA as a function of frequency. MUA power during flexion was significantly larger than during extension (two-sample *t* test, α = 0.01, *n* = 2,494 MUA power comparisons).

Figure 7*A* presents the comparisons between the MUA power spectra during the flexion and extension phases grouped per lumbar segment across frequencies. Bars indicate the segment and frequency bands where the power spectrum during a phase of stepping was significantly greater than during the other phase. For the vast majority of locations and frequencies, power was greater during the flexion phase. The frequency bins with significantly greater MUA power were summed across all lumbosacral locations and the resulting histogram is presented in Fig. 7*B* While areas of greater MUA power during extension occur in the 7- to 12- and 28- to 41-Hz frequency bands, there was greater MUA power during flexion across all frequencies.

In total, there were 2,494 MUA power comparisons from 58 electrode arrays (2 per trial) and 43 frequency bands (between 7 and 50 Hz) (2,494 squares in Fig. 7*A*, *top*). Two-group hypothesis testing showed that the multiunit activity was greater during flexion in 855/2,494 (34%) comparisons, greater during extension in 62/2,494 (3%) comparisons and equitable between conditions in 1,577/2,494 (63%) comparisons. Overall, the results indicate that MUA power is greater during the flexion phase.

### Multiunit Coherence between Segments

**Figure 8.**
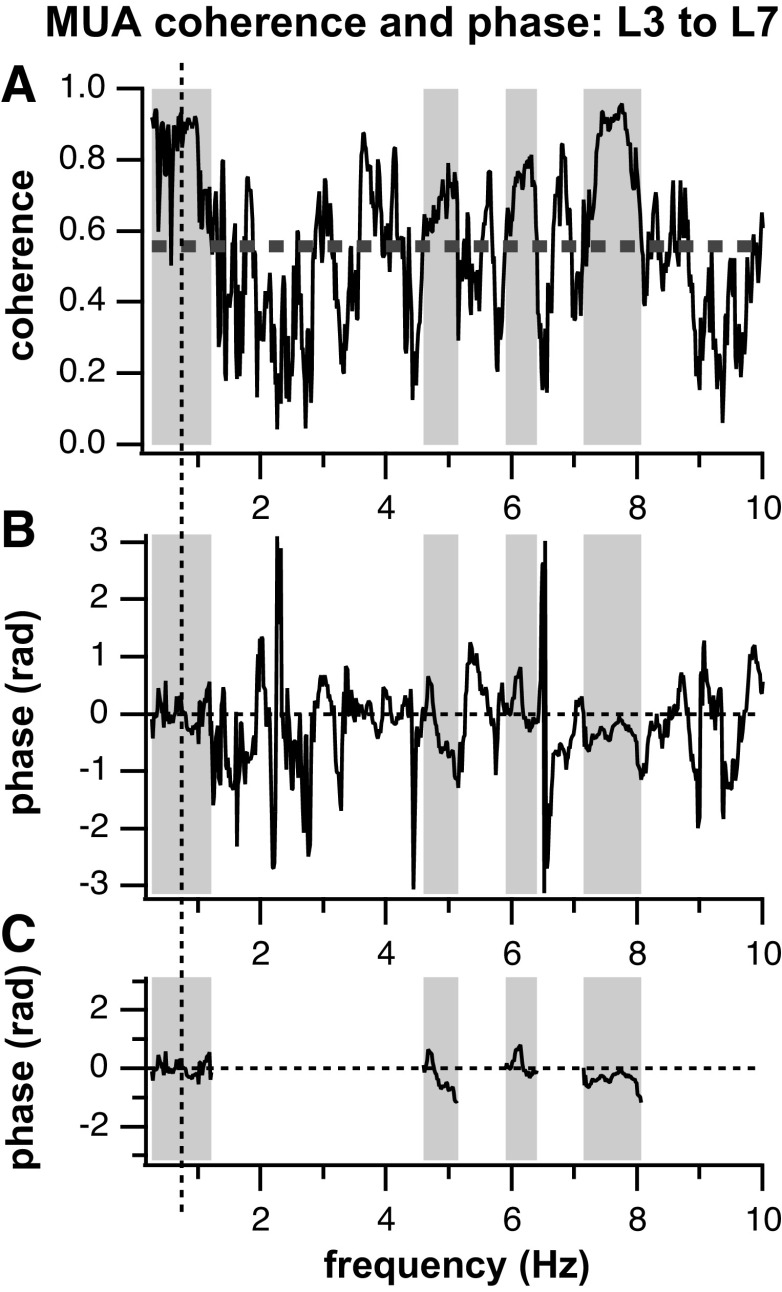
Example of coherence and phase between multiunit activity (MUA) activity of L3 and L7 segments of Fig. 2. *A*: coherence for frequencies from 0.25 to 10 Hz (higher frequency coherence from 10 to 50 Hz not shown). Dotted horizontal line is the 95% confidence interval for significant coherence. Gray shading indicates frequency bands where coherence is greater than a 95% confidence interval for greater than 24 consecutive samples (0.37 Hz). *B*: corresponding phase difference between the L3 and L7 averaged MUA activity. *C*: phase difference between L3 and L7 MUA activity at the frequencies of significant coherence. The vertical dotted line in *A*–*C* indicates the average frequency of stepping for the trial.

**Figure 9.**
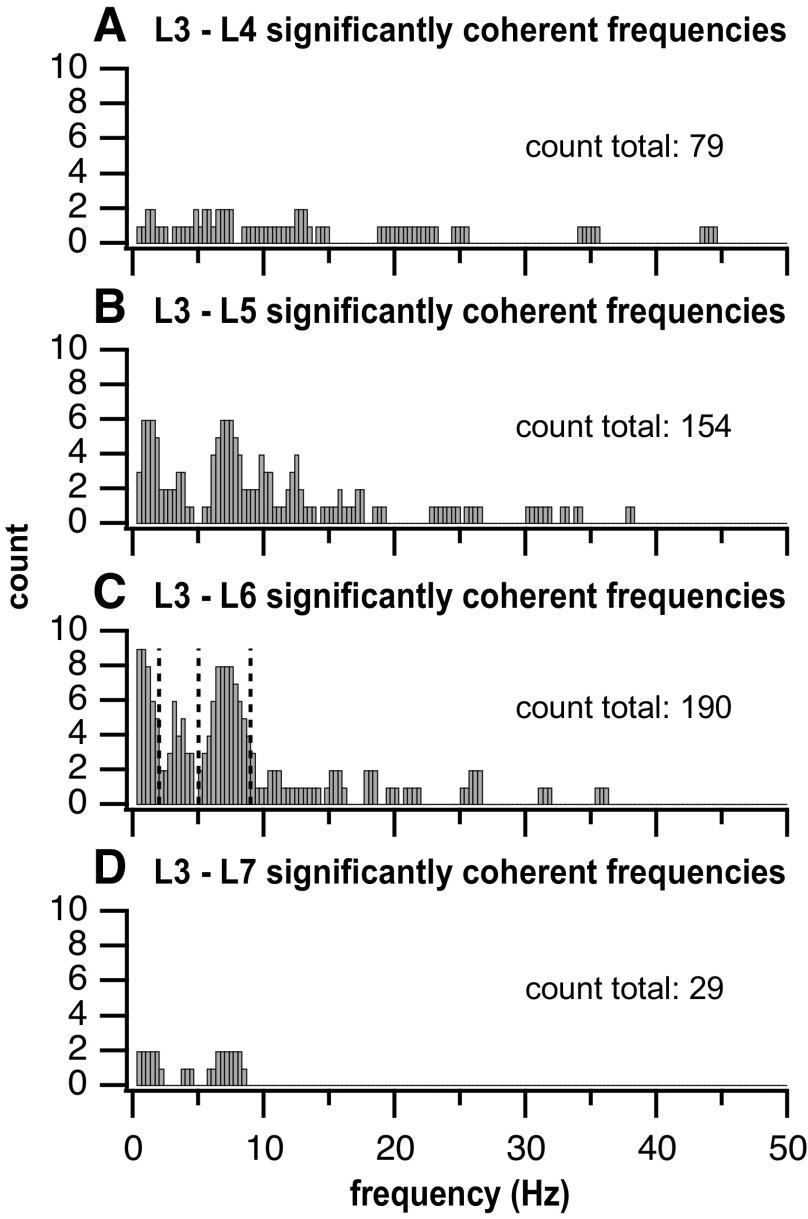
Histogram of the counts of frequency bins with significant coherence across all subjects and rostrocaudal segment separations. Rostrocaudal separation from L3 increases from *A* to *D*, i.e, L3 to L4 (*A*), L3 to L5 (*B*), L3 to L6 (*C*), and L3 to L7 (*D*). We observed 3 frequency band peaks in the histogram, most apparent for the L3–L6 separation: 0.25–2 Hz, 2–5 Hz, and 5–9 Hz. Vertical lines in *C* indicate the 2-, 5-, and 9-Hz frequencies.

Twenty-four air-stepping trials were analyzed for coherence and phase between MUA at different segments (see Fig. 8 for an example analysis). These trials were recorded from four subjects (*subject 1*: 2; *subject 2*: 8; *subject 3*: 4; and *subject 4*: 10). Overall, 30 unique recording locations were processed among the 4 subjects. Trials per rostrocaudal lumbar segment separations varied: 2 trials for L3–L4, 8 trials for L3–L5, 10 trials for L3–L6, and 4 trials for L3–L7. The majority of significantly coherent frequencies fell into low (0.25–2 Hz)-, mid (2–5 Hz)-, and high (5–9 Hz)-frequency bands across all segmental separations (Fig. 9).

**Figure 10.**
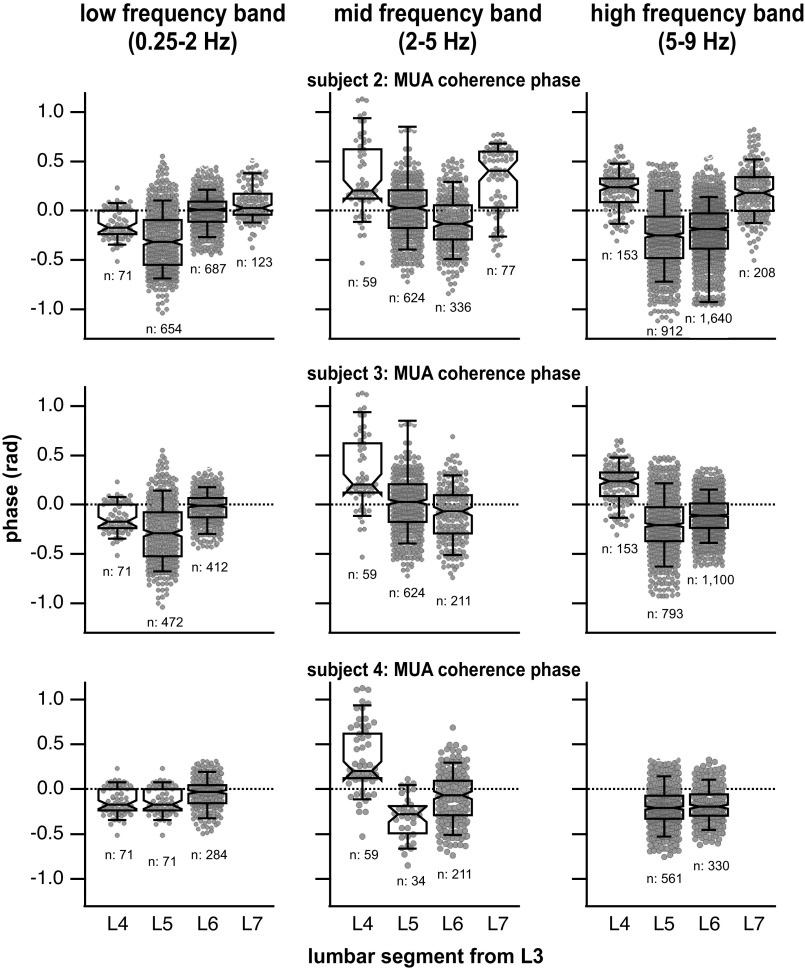
Multiunit activity (MUA) coherence phase per subject: Box plots of the phases between MUA activity at L3 and consecutive lumbar segments (± 3.14 rad = 180° phase difference; ± 0.1 rad = 5.7° phase difference). The phase differences’ mean and standard deviations are no more than 0.4 rad in any instance indicating a progression of activation on a time scale of axonal/synaptic transmission and therefore synchronous activation across lumbar segments for all frequency bands. Whiskers indicate the 9th and 91st percentiles.

Figure 10 shows the phase differences between the MUA activity recorded at different lumbar segments for each subject and frequency band. A few trends appear *1*) all mean and median phase differences are relatively close to zero, with the greatest deviation from zero phase difference being ∼0.4 rad, and *2*) the values of the phase differences between segments are inconsistent with no indication of an increase in phase with the distance between segments. The only consistent results are the very small values of the phase differences, no larger than 0.4 rad and typically much less, between L3 and caudal segments.

Assuming that the phase measurements occur within one cycle period, the time associated with a phase difference of 0.15 rad in the low-frequency band (∼1 Hz) is ∼24 ms. For the high-frequency band (∼7 Hz) and a maximum phase difference of 0.4 rad, the time difference in activation between segments is ∼9 ms. For a lumbar enlargement distance of 35 mm, these times indicate conduction velocity between roughly 1.5 and 3.9 m/s (assuming no synapses in between). These implications will be further discussed in the discussion.

## DISCUSSION

### Summary

This study analyzed spinal multiunit signals during stepping for evidence of differences in activity per lumbar segment and phases of locomotion and to characterize the patterns of neural activation along the lumbar enlargement. Consistent with previous studies of the spinal rhythmic generation centers in the cat (11, 12, 57), we found more multiunit power in midlumbar segments L3–L5 during stepping than in more caudal segments. We also found significantly greater multiunit power during the flexion phase of stepping than during the extension phase of stepping for all lumbar segments. Multiunit activity power levels may represent degrees of overall neural activity during stepping and the greater power during flexion matches previously reported asymmetries between flexor- and extensor-related population firing of the spinal locomotor networks (26, 27). Finally, the multiunit power showed no phase lag at coherent frequencies throughout the lumbar enlargement indicative of a longitudinal standing wave model for MUA activity. Taken together, these results suggest that MUA activity may represent the output of a rostrocaudally synchronized flexor dominant rhythmic-generating center.

### Spectral Power of Spinal Interneuron Multiunit Activity

#### Rostrocaudal multiunit power differences.

Various studies have concluded that the L3–L5 lumbar segments are essential for rhythmic alternation of the hindlimbs in the cat (11, 12), while further caudal segments show less robust rhythm-generating capability (57).

Our results are somewhat supportive of the hypothesis that the rhythm-generating centers are located in L3–L5, especially when considering the multiunit power distribution across lumbar segments for the frequency bands with peaks in the multiunit power spectrum (low, 0.5–3 Hz; high, 6–10 Hz) (Fig. 5). While the lumbar segments of greatest multiunit power differ slightly between these two bands (Fig. 5) (L4–L5 in the low band and L3–L4 in the high band), the greatest MUA power overall is in the midlumbar segments L3–L5.

#### Differences in multiunit power per phase of stepping.

In McMahon et al. (41), we presented evidence that two ensembles of interneurons within the lumbar enlargement encode for opposing phases of the step cycle (Fig. 6 of Ref. 41) with a similar number of units tuned to the flexor or extensor phase (Fig. 5 of Ref. 41). We therefore anticipated that the multiunit activity would reflect the activity of the isolated single units and show similar power during flexion and extension. However, contrary to this hypothesis, our results demonstrated greater power during flexion than extension across all lumbar segments. This result supports previous observations of a difference in the rhythmic drive to flexor and extensor motor neurons during stepping (26, 27).

The difference in the information supplied by the continuous multiunit signals and the discrete spike times of single units may explain the contradictory results of unbalanced MUA power between the flexion and extension phases and comparable distribution of extensor and flexor-tuned ensembles. While discrete spike time data are known to be biased toward the local, large amplitude spiking neurons, the continuous multiunit activity reflects the changes in the magnitude of extracellular potentials. These extracellular potentials include the summation of both large-amplitude action potentials from large, local cells as well as synchronously active small action potentials from smaller/farther neurons (3). Thus the electrical potential of nonspiking cells and of small, synchronous cells may be represented in the continuous multiunit activity but absent from the individual spike times of spinal interneurons within the same locations.

The absence of load-bearing feedback in our preparation may contribute to the reduced power during extension, but other locomotor preparations suggest that a flexor-dominant CPG may be due to differences in the central locomotor circuit, independent of modifications or the absence of particular sensory feedback. For example, there was a complete absence of muscle afferents during fictive locomotion observations of nonresetting deletions (27) that led to the same hypothesis of a flexor-dominant rhythm center.

In studies of mesencephalic locomotor region-stimulated fictive locomotion in the cat, Yakovenko et al. (58) designated the locomotor phase with the largest variations in timing as the “dominant” phase. In this study, changes in cycle duration were attributed to larger variations in the flexor phase in 71% of the cases, implying flexor dominance. The authors also observed that either the extensor or flexor burst was active for more than half of the step cycle and suggested that it was actually the longer muscle burst duration that indicated flexor or extensor dominance. Based on these findings, the authors hypothesize that the central pattern generator is not flexor or extensor biased but is instead modulated by sensory input. The lack of sensory feedback in the fictive locomotion preparation would result in flexor dominance of the rhythm-generating level of the CPG.

In our study, the onset and offset times of muscle bursts grouped evenly by phase (flexor or extensor) with very little variance per subject (Fig. 3 of Ref. 41) and the 95% confidence interval of the flexor onset phase within the right step was between 60 and 64% of a step (3.8–4 rad) for all subjects. Furthermore, the extension burst lasted ∼50% of the step cycle while the flexion burst lasted <40% of the step cycle in all cases. If we were to classify the dominant phase of the rhythm generator based on the muscle burst duration, we would hypothesize an extensor-dominated CPG, which is contrary to our findings of greater multiunit power during the flexion phase of stepping.

One major caveat of our analysis is that because of the durations of the flexion and extension phases we could not resolve power at frequencies below 7 Hz, which precluded us from obtaining the power during the flexor and extensor phases at the actual frequency of stepping (≈1 Hz). Although power was consistently higher during the flexor phase than the extensor phase for the 7- to 50-Hz frequency band (see Fig. 3), we cannot totally exclude the possibility that the MUA activity spectrum would reverse and show greater power during the extensor phase at the frequency of stepping), although that would be very unlikely. A fundamental at around 1 Hz with greater power in extension than flexion would produce harmonics with greater power in extension than flexion at multiples of the stepping frequency and we don’t see that phenomenon, especially for the more rostral segments.

#### Multiunit activation patterns along the lumbar enlargement.

Three patterns of neural activation along the lumbar cord during rhythmic behavior have been proposed: the traveling wave, the standing wave, and the modular organization. The traveling wave, as seen in fictive scratching, shows a rostrocaudal progression of activation from L4 to S1 during a 3-Hz scratching rhythm that lasts approximately a third of the scratch cycle (∼1.9 rad) (33, 59). If the spinal interneurons of lamina V–VII were activated in a traveling wave progression, we would expect to see a similar phase difference of ∼1.9 rad in MUA activity from L3 to L7 within the low-frequency band. The phase differences observed were much lower (∼0.4 rad) for our MUA signals (Fig. 10).

A longitudinal standing wave of activation displayed no relation between rostrocaudal position and peak of multiunit neural activation in the air-stepping cat (35). The multiunit activity had maximal activity on the transition from swing to stance and the duration of the longitudinal standing wave activation was on the order of a step cycle (∼1,000 ms). In another study in the cat, synchronous intersegmental patterns of neuronal firing during spontaneous negative cord dorsum potentials were measured on a timescale of ∼25 ms (60). This study found latency shifts with L5 and L6 activity preceding L4 and L7 activity by 5.8 and 9.0 ms. While these authors suggest that these shifts could indicate the propagation of signals from one segment to another, they also may result from differences in conduction time from a common input to different segments.

While a nonmonotonic progression of activation has not, to our knowledge, been demonstrated in spinal interneuronal systems, the progression of activation through motor pools has been described in this fashion in humans and cats (38, 39). The center of activation or peak of MUA activity is seen to rapidly shift from midlumbar segments during flexion (∼30% of a step cycle, caudal L4–rostral L6) to caudal lumbar segments during extension (∼50% of a step cycle, caudal L5–rostral S1) in a span of ∼10% of the step cycle. If this shift of activation was present within the low-frequency band for the population of spinal interneurons from which we recorded, we should have seen little to no phase difference in MUA coherence phase between L3 and L5 segments (MUA in phase within those segments) and then a large phase difference of ∼3.1 rad (out of phase MUA activity) between L3 and L6 as well as between L3 and L7, differences that we did not observe (see Fig. 10).

For any of these proposed modes of progression of the neural activation along the lumbar cord, it is important to define a specified time scale for phenomena like “shifts in activity” and “synchrony.” Neural conduction velocity within the spinal cord typically ranges from ∼20 to 60 m/s (61–63). For a 35-mm span of the lumbar enlargement, this translates to between 1.6 and 3 ms for a disynaptic connection (assuming a 0.5-ms delay per synapse) between neurons of L3 and L7. In contrast to these very short time scales of neural transmission, the duration of the rhythmic movements discussed here such as scratch, swimming, and locomotion range from 250 to 2,000 ms per cycle. This time scale is two to four orders of magnitude greater than synaptic transmission. We are defining synchrony on the time scale of neural transmission through a few synapses (i.e., tens of milliseconds, compared to an ∼1,000-ms step duration). In addition to varying differently across subjects, our phase differences between segments were very low and we attribute them to neural transmission delays involving very few synapses along the rostrocaudal spinal cord. We believe that a small time difference between MUA activation along the cord is in fact an indication of synchrony in distributed spinal interneurons, indicative of a longitudinal standing wave of activation.

## DATA AVAILABILITY

Data will be made available upon reasonable request.

## GRANTS

This study was funded by National Institutes of Health Grants NS110605, NS055976, and EB012855.

## DISCLAIMERS

The funding sources had no involvement in study design, data collection, analysis, interpretation, or reporting.

## DISCLOSURES

No conflicts of interest, financial or otherwise, are declared by the authors.

## AUTHOR CONTRIBUTIONS

C.M., D.P.K., A.J.K., and M.A.L. conceived and designed research; C.M., D.P.K., A.J.K., and M.A.L. performed experiments; C.M., D.P.K., and M.A.L. analyzed data; C.M., D.P.K., and M.A.L. interpreted results of experiments; C.M., D.P.K., and M.A.L. prepared figures; C.M. and D.P.K. drafted manuscript; C.M., D.P.K., A.J.K., and M.A.L. edited and revised manuscript; C.M., D.P.K., A.J.K., and M.A.L. approved final version of manuscript.

## References

[1] Burns SP, Xing D, Shapley RM. Comparisons of the dynamics of local field potential and multiunit activity signals in macaque visual cortex. J Neurosci 30: 13739–13749, 2010. doi:10.1523/JNEUROSCI.0743-10.2010. 20943914 PMC3518461

[2] Buzsaki G. Large-scale recording of neuronal ensembles. Nat Neurosci 7: 446–451, 2004. doi:10.1038/nn1233. 15114356

[3] Logothetis NK. The underpinnings of the BOLD functional magnetic resonance imaging signal. J Neurosci 23: 3963–3971, 2003. doi:10.1523/JNEUROSCI.23-10-03963.2003. 12764080 PMC6741096

[4] Amjad AM, Halliday DM, Rosenberg JR, Conway BA. An extended difference of coherence test for comparing and combining several independent coherence estimates: theory and application to the study of motor units and physiological tremor. J Neurosci Methods 73: 69–79, 1997. doi:10.1016/s0165-0270(96)02214-5. 9130680

[5] Baker SN, Pinches EM, Lemon RN. Synchronization in monkey motor cortex during a precision grip task. II. effect of oscillatory activity on corticospinal output. J Neurophysiol 89: 1941–1953, 2003. doi:10.1152/jn.00832.2002. 12686573

[6] Conway BA, Halliday DM, Farmer SF, Shahani U, Maas P, Weir AI, Rosenberg JR. Synchronization between motor cortex and spinal motoneuronal pool during the performance of a maintained motor task in man. J Physiol 489: 917–924, 1995. doi:10.1113/jphysiol.1995.sp021104. 8788955 PMC1156860

[7] Miller WL, Sigvardt KA. Spectral analysis of oscillatory neural circuits. J Neurosci Methods 80: 113–128, 1998. doi:10.1016/s0165-0270(97)00185-4. 9667384

[8] Mor Y, Lev-Tov A. Analysis of rhythmic patterns produced by spinal neural networks. J Neurophysiol 98: 2807–2817, 2007. doi:10.1152/jn.00740.2007. 17715187

[9] Kwan AC, Dietz SB, Zhong G, Harris-Warrick RM, Webb WW. Spatiotemporal dynamics of rhythmic spinal interneurons measured with two-photon calcium imaging and coherence analysis. J Neurophysiol 104: 3323–3333, 2010. doi:10.1152/jn.00679.2010. 20861442 PMC3007658

[10] Nielsen JB, Conway BA, Halliday DM, Perreault MC, Hultborn H. Organization of common synaptic drive to motoneurones during fictive locomotion in the spinal cat. J Physiol 569: 291–304, 2005. doi:10.1113/jphysiol.2005.091744. 16166163 PMC1464221

[11] Deliagina TG, Orlovsky GN, Pavlova GA. The capacity for generation of rhythmic oscillations is distributed in the lumbosacral spinal cord of the cat. Exp Brain Res 53: 81–90, 1983. doi:10.1007/BF00239400. 6674000

[12] Langlet C, Leblond H, Rossignol S. Mid-lumbar segments are needed for the expression of locomotion in chronic spinal cats. J Neurophysiol 93: 2474–2488, 2005. doi:10.1152/jn.00909.2004. 15647400

[13] Cazalets JR, Borde M, Clarac F. Localization and organization of the central pattern generator for hindlimb locomotion in newborn rat. J Neurosci 15: 4943–4951, 1995. doi:10.1523/JNEUROSCI.15-07-04943.1995. 7623124 PMC6577873

[14] Cowley KC, Schmidt BJ. Regional distribution of the locomotor pattern-generating network in the neonatal rat spinal cord. J Neurophysiol 77: 247–259, 1997. doi:10.1152/jn.1997.77.1.247. 9120567

[15] Ho S, O'Donovan MJ. Regionalization and intersegmental coordination of rhythm-generating networks in the spinal cord of the chick embryo. J Neurosci 13: 1354–1371, 1993. doi:10.1523/JNEUROSCI.13-04-01354.1993. 8463824 PMC6576707

[16] Kjaerulff O, Kiehn O. Distribution of networks generating and coordinating locomotor activity in the neonatal rat spinal cord in vitro: a lesion study. J Neurosci 16: 5777–5794, 1996. doi:10.1523/JNEUROSCI.16-18-05777.1996. 8795632 PMC6578971

[17] Mortin LI, Stein PS. Spinal cord segments containing key elements of the central pattern generators for three forms of scratch reflex in the turtle. J Neurosci 9: 2285–2296, 1989. doi:10.1523/JNEUROSCI.09-07-02285.1989. 2746329 PMC6569757

[18] Wheatley M, Jovanovic K, Stein RB, Lawson V. The activity of interneurons during locomotion in the in vitro necturus spinal cord. J Neurophysiol 71: 2025–2032, 1994. doi:10.1152/jn.1994.71.6.2025. 7931500

[19] McCrea DA, Rybak IA. Organization of mammalian locomotor rhythm and pattern generation. Brain Res Rev 57: 134–146, 2008. doi:10.1016/j.brainresrev.2007.08.006. 17936363 PMC2214837

[20] Martinez M, Tuznik M, Delivet-Mongrain H, Rossignol S. Emergence of deletions during treadmill locomotion as a function of supraspinal and sensory inputs. J Neurosci 33: 11599–11605, 2013. doi:10.1523/JNEUROSCI.1126-13.2013. 23843528 PMC6618683

[21] Rybak IA, Shevtsova NA, Lafreniere-Roula M, McCrea DA. Modelling spinal circuitry involved in locomotor pattern generation: insights from deletions during fictive locomotion. J Physiol 577: 617–639, 2006. doi:10.1113/jphysiol.2006.118703. 17008376 PMC1890439

[22] Linden H, Petersen PC, Vestergaard M, Berg RW. Movement is governed by rotational neural dynamics in spinal motor networks. Nature 610: 526–531, 2022. doi:10.1038/s41586-022-05293-w. 36224394

[23] Baldissera F, Hultborn H, Illert M. Integration in spinal neuronal systems. In: Handbook of Physiology, The Nervous System Motor Control. Bethesda, MD: American Physiology Society, 1981, p. 509–595.

[24] Eccles RM, Lundberg A. Synaptic actions in motoneurones by afferents which may evoke the flexion reflex. Arch Ital Biol 97: 199–221, 1959.

[25] Burke RE, Degtyarenko AM, Simon ES. Patterns of locomotor drive to motoneurons and last-order interneurons: clues to the structure of the CPG. J Neurophysiol 86: 447–462, 2001. doi:10.1152/jn.2001.86.1.447. 11431524

[26] Pearson KG, Duysens J. Function of segmental reflexes in the control of stepping in cockroaches and cats. In: Neural Control of Locomotion, edited by Herman RM, Grillner S, Stein PS. Boston, MA: Springer, 1976, p. 519–537.

[27] Zhong G, Shevtsova NA, Rybak IA, Harris-Warrick RM. Neuronal activity in the isolated mouse spinal cord during spontaneous deletions in fictive locomotion: insights into locomotor central pattern generator organization. J Physiol 590: 4735–4759, 2012. doi:10.1113/jphysiol.2012.240895. 22869012 PMC3487034

[28] Duysens J, De Groote F, Jonkers I. The flexion synergy, mother of all synergies and father of new models of gait. Front Comput Neurosci 7: 14, 2013. doi:10.3389/fncom.2013.00014. 23494365 PMC3595503

[29] Sigvardt KA, Miller WL. Analysis and modeling of the locomotor central pattern generator as a network of coupled oscillators. Ann N Y Acad Sci 860: 250–265, 1998. doi:10.1111/j.1749-6632.1998.tb09054.x. 9928317

[30] Stein PSG. Neural control of interappendage phase during locomotion. Am Zool 14: 1003–1016, 1974. doi:10.1093/icb/14.3.1003.

[31] Wiggin TD, Anderson TM, Eian J, Peck JH, Masino MA. Episodic swimming in the larval zebrafish is generated by a spatially distributed spinal network with modular functional organization. J Neurophysiol 108: 925–934, 2012. doi:10.1152/jn.00233.2012. 22572943 PMC3424096

[32] Cuellar CA, Trejo A, Linares P, Delgado-Lezama R, Jimenez-Estrada I, Abyazova LM, Baltina TV, Manjarrez E. Spinal neurons bursting in phase with fictive scratching are not related to spontaneous cord dorsum potentials. Neuroscience 266: 66–79, 2014. doi:10.1016/j.neuroscience.2014.02.003. 24530658

[33] Perez T, Tapia JA, Mirasso CR, Garcia-Ojalvo J, Quevedo J, Cuellar CA, Manjarrez E. An intersegmental neuronal architecture for spinal wave propagation under deletions. J Neurosci 29: 10254–10263, 2009. doi:10.1523/JNEUROSCI.1737-09.2009. 19692599 PMC6665792

[34] Siegel M, Donner TH, Engel AK. Spectral fingerprints of large-scale neuronal interactions. Nat Rev Neurosci 13: 121–134, 2012. doi:10.1038/nrn3137. 22233726

[35] AuYong N, Ollivier-Lanvin K, Lemay MA. Population spatiotemporal dynamics of spinal intermediate zone interneurons during air-stepping in adult spinal cats. J Neurophysiol 106: 1943–1953, 2011. doi:10.1152/jn.00258.2011. 21775722 PMC3191843

[36] AuYong N, Ollivier-Lanvin K, Lemay MA. Preferred locomotor phase of activity of lumbar interneurons during air-stepping in subchronic spinal cats. J Neurophysiol 105: 1011–1022, 2011. doi:10.1152/jn.00523.2010. 21084683 PMC3074417

[37] Chavez D, Rodriguez E, Jimenez I, Rudomin P. Changes in correlation between spontaneous activity of dorsal horn neurones lead to differential recruitment of inhibitory pathways in the cat spinal cord. J Physiol 590: 1563–1584, 2012. doi:10.1113/jphysiol.2011.223271. 22271870 PMC3413496

[38] Cappellini G, Ivanenko YP, Dominici N, Poppele RE, Lacquaniti F. Migration of motor pool activity in the spinal cord reflects body mechanics in human locomotion. J Neurophysiol 104: 3064–3073, 2010. doi:10.1152/jn.00318.2010. 20881204

[39] Yakovenko S, Mushahwar V, VanderHorst V, Holstege G, Prochazka A. Spatiotemporal activation of lumbosacral motoneurons in the locomotor step cycle. J Neurophysiol 87: 1542–1553, 2002. doi:10.1152/jn.00479.2001. 11877525

[40] Abeles M, Hayon G, Lehmann D. Modeling compositionality by dynamic binding of synfire chains. J Comput Neurosci 17: 179–201, 2004. doi:10.1023/B:JCNS.0000037682.18051.5f. 15306739

[41] McMahon C, Kowalski DP, Krupka AJ, Lemay MA. Single-cell and ensemble activity of lumbar intermediate and ventral horn interneurons in the spinal air-stepping cat. J Neurophysiol 127: 99–115, 2022. doi:10.1152/jn.00202.2021. 34851739 PMC8721903

[42] Krupka AJ, Fischer I, Lemay MA. Transplants of neurotrophin-producing autologous fibroblasts promote recovery of treadmill stepping in the acute, sub-chronic, and chronic spinal cat. J Neurotrauma 34: 1858–1872, 2016. doi:10.1089/neu.2016.4559. 27829315 PMC5444492

[43] Jinks SL, Atherley RJ, Dominguez CL, Sigvardt KA, Antognini JF. Isoflurane disrupts central pattern generator activity and coordination in the lamprey isolated spinal cord. Anesthesiology 103: 567–575, 2005. doi:10.1097/00000542-200509000-00020. 16129982

[44] McCrea DA, Shefchyk SJ, Stephens MJ, Pearson KG. Disynaptic group I excitation of synergist ankle extensor motoneurones during fictive locomotion in the cat. J Physiol 487: 527–539, 1995. doi:10.1113/jphysiol.1995.sp020897. 8558481 PMC1156590

[45] Micera S, Sabatini AM, Dario P. An algorithm for detecting the onset of muscle contraction by EMG signal processing. Med Eng Phys 20: 211–215, 1998. doi:10.1016/s1350-4533(98)00017-4. 9690491

[46] Stark E, Abeles M. Predicting movement from multiunit activity. J Neurosci 27: 8387–8394, 2007. doi:10.1523/JNEUROSCI.1321-07.2007. 17670985 PMC6673077

[47] Legatt AD, Arezzo J, Vaughan HG. Jr. Averaged multiple unit activity as an estimate of phasic changes in local neuronal activity: effects of volume-conducted potentials. J Neurosci Methods 2: 203–217, 1980. doi:10.1016/0165-0270(80)90061-8. 6771471

[48] Bokil H, Andrews P, Kulkarni JE, Mehta S, Mitra PP. Chronux: a platform for analyzing neural signals. J Neurosci Methods 192: 146–151, 2010. doi:10.1016/j.jneumeth.2010.06.020. 20637804 PMC2934871

[49] Bokil H, Purpura K, Schoffelen JM, Thomson D, Mitra P. Comparing spectra and coherences for groups of unequal size. J Neurosci Methods 159: 337–345, 2007. doi:10.1016/j.jneumeth.2006.07.011. 16945422

[50] Mitra P, Bokil H. Observed Brain Dynamics. Oxford, UK: Oxford University Press, 2008.

[51] Zeitler M, Fries P, Gielen S. Assessing neuronal coherence with single-unit, multi-unit, and local field potentials. Neural Comput 18: 2256–2281, 2006. doi:10.1162/neco.2006.18.9.2256. 16846392

[52] Thissen D, Steinberg L, Kuang D. Quick and easy implementation of the Benjamini-Hochberg procedure for controlling the false positive rate in multiple comparisons. J Educ Behav Stat 27: 77–83, 2002. doi:10.3102/10769986027001077.

[53] Benjamini Y, Hochberg Y. Controlling the false discovery rate: a practical and powerful approach to multiple testing. J Royal Stat Soc B 57: 289–300, 1995. doi:10.1111/j.2517-6161.1995.tb02031.x.

[54] Goldfine AM, Victor JD, Conte MM, Bardin JC, Schiff ND. Determination of awareness in patients with severe brain injury using EEG power spectral analysis. Clin Neurophysiol 122: 2157–2168, 2011. doi:10.1016/j.clinph.2011.03.022. 21514214 PMC3162107

[55] Schoffelen JM, Oostenveld R, Fries P. Neuronal coherence as a mechanism of effective corticospinal interaction. Science 308: 111–113, 2005. doi:10.1126/science.1107027. 15802603

[56] Raethjen J, Lindemann M, Dumpelmann M, Wenzelburger R, Stolze H, Pfister G, Elger CE, Timmer J, Deuschl G. Corticomuscular coherence in the 6-15 Hz band: is the cortex involved in the generation of physiologic tremor? Exp Brain Res 142: 32–40, 2002. doi:10.1007/s00221-001-0914-7. 11797082

[57] Grillner S, Zangger P. On the central generation of locomotion in the low spinal cat. Exp Brain Res 34: 241–261, 1979. doi:10.1007/BF00235671. 421750

[58] Yakovenko S, McCrea DA, Stecina K, Prochazka A. Control of locomotor cycle durations. J Neurophysiol 94: 1057–1065, 2005. doi:10.1152/jn.00991.2004. 15800075

[59] Cuellar CA, Tapia JA, Juarez V, Quevedo J, Linares P, Martinez L, Manjarrez E. Propagation of sinusoidal electrical waves along the spinal cord during a fictive motor task. J Neurosci 29: 798–810, 2009. doi:10.1523/JNEUROSCI.3408-08.2009. 19158305 PMC6665157

[60] Manjarrez E, Jimenez I, Rudomin P. Intersegmental synchronization of spontaneous activity of dorsal horn neurons in the cat spinal cord. Exp Brain Res 148: 401–413, 2003. doi:10.1007/s00221-002-1303-6. 12541150

[61] Alstermark B, Kummel H, Pinter MJ, Tantisira B. Integration in descending motor pathways controlling the forelimb in the cat. 17. Axonal projection and termination of C3-C4 propriospinal neurones in the C6-Th1 segments. Exp Brain Res 81: 447–461, 1990. doi:10.1007/BF02423494. 2226681

[62] Blight AR. Axonal physiology of chronic spinal cord injury in the cat: intracellular recording in vitro. Neuroscience 10: 1471–1486, 1983. doi:10.1016/0306-4522(83)90128-8. 6664497

[63] Xi MC, Liu RH, Engelhardt JK, Morales FR, Chase MH. Changes in the axonal conduction velocity of pyramidal tract neurons in the aged cat. Neuroscience 92: 219–225, 1999. doi:10.1016/s0306-4522(98)00754-4. 10392844

